# Longitudinal Associations of (Un)popularity with Weight Perceptions and Dieting in Adolescence

**DOI:** 10.1007/s10964-024-02090-8

**Published:** 2024-09-25

**Authors:** Aafke Swinkels, Nina van den Broek, Antonius H. N. Cillessen

**Affiliations:** Behavioural Science Institute, Radboud University, P.O. Box 9104, 6500 HE Nijmegen, The Netherlands

**Keywords:** Popularity, Liking, Self-esteem, Weight perception, Dieting

## Abstract

Little is known about the unique effects of (un)popularity on body image and the characteristics influencing these effects. The goals of this study were to examine (1) the longitudinal associations of adolescents’ (un)popularity with weight perception and dieting, (2) whether (dis)liking, self-esteem, and gender moderated these associations. Participants were 1697 Dutch adolescents (*M*_age_ = 14.18 years, *SD* = 1.29; 51% female), from a middle-class population. Participants completed peer nominations and self-reports in three consecutive school years. Mixed-effects models showed that (un)popularity did not predict weight perception and dieting over time. Concurrently, when liking was low, popularity predicted positive weight perception. Higher popularity predicted more dieting in females. This study highlighted that adolescents’ body image varied in subgroups of social status.

## Introduction

The number of adolescents with a negative body image and who engage in harmful dieting is alarmingly high. *Body image* is an umbrella term for how individuals think, feel, perceive, and act towards their body. Many adolescents perceive themselves as too fat, and this percentage increases with age. In the Netherlands, 33% of females and 23% of males at age 11 and 51% of females and 27% of males at age 15 perceived themselves as too fat (World Health Organization, 2015). In the United States, 45% of high school-aged students reported trying to lose weight, and 23% reported unhealthy dieting strategies such as vomiting, using laxatives, and fasting (CDC, 2006). Adolescents (particularly females) also diet when they are not overweight or even underweight (CDC, 2006; Huon & Lim, 2000). *Dieting* can indicate how eagerly adolescents want to change their body. Dieting (if done appropriately) can also be beneficial for adolescents who are truly overweight, but it is here interpreted as a risk for unhealthy eating behavior (Allen et al., 2014). Because negative weight perception and unhealthy dieting in adolescence can cause health problems later in life (Barakat et al., 2023; Goldschmidt et al., 2016), it is important to understand their development. An important factor that influences adolescents’ weight perception and dieting is their social status in the peer group (Schwartz & Gorman; Webb & Zimmer-Gembeck, 2014), but research on the effects of peer status on weight-related perceptions and behaviors is limited. The current study addressed this research gap by examining in detail the associations of adolescents’ popularity and unpopularity in the peer group with their weight perceptions and dieting, both concurrently and longitudinally.

Peers play an important role in the development of adolescents’ body image (Webb & Zimmer-Gembeck, 2014). Research has shown that adolescents’ body image can be influenced by peers who shape beliefs about the ideal body (Reel et al., 2015). Adolescents’ social status plays a role in the degree to which they are influenced by their peers (Schwartz & Gorman, 2011). Having an “ideal body” is a social norm (Reel et al., 2015), and popular adolescents focus more than other youth on adhering to social norms (Cillessen et al., 2011; Valente et al., 2005). As a result, they may have a more ideal body and a more positive weight perception than other adolescents. Popular adolescents may focus more on their appearance and weight than other adolescents and therefore engage in more (unhealthy) dieting to meet social expectations of the ideal body. As popular adolescents are known to engage in health risk behaviors (Cillessen et al., 2023), they may also engage in “risky” or unhealthy dieting.

In contrast, unpopular adolescents tend to behave in ways that deviate from the norms of the peer group (Schwartz & Gorman, 2011). It has been found that the body shape of unpopular adolescents is on average less “ideal” (e.g., Wang et al., 2006). Unpopularity predicts victimization (Gorman et al., 2011) and appearance victimization increases body dissatisfaction (Webb & Zimmer-Gembeck, 2014). Thus, unpopular adolescents might have a negative weight perception and therefore might try to change their weight by dieting, while popular adolescents might have a more positive weight perception, but still diet to adhere to group norms.

Only three studies have examined the associations of popularity with measures of body image. Adolescents high in popularity generally had lower body dissatisfaction (Rancourt & Prinstein, 2010), but also focused more on attaining the ideal body (Rancourt & Prinstein, 2010; Wang et al., 2006). In contrast, in another study only adolescents low in popularity had increased risk for disordered eating (Smink et al., 2018). Given these mixed results, more research is needed to understand the association between popularity and dieting.

The three previous studies treated popularity as a single continuum, and operationalized unpopularity as ‘low popularity’. In sociometric research, it is common to create composite scores (most popular minus least popular; liked most minus liked least; Stoltz et al., 2016). However, there may be unique effects of popularity versus unpopularity. Considering popularity and unpopularity as separate constructs allows for a broader understanding of the role of peer group status in status-related cognitions and behaviors (Gorman et al., 2011), such as weight perception and dieting. In a study that looked at popularity and unpopularity as separate predictors, Gorman et al. (2011) found that unpopularity uniquely predicted victimization, withdrawal, prosociality, and loneliness, while popularity uniquely predicted aggression. This implies that unpopularity is distinct from low levels of popularity. It is expected that unpopularity would predict negative weight perceptions and more dieting, while popularity would predict positive weight perceptions but also more dieting. Separating popularity from unpopularity may significantly contribute to the understanding of adolescents’ weight perception and dieting.

### Moderators

Beyond the main effects of popularity and unpopularity for prevention and intervention efforts, it is of utmost important to identify characteristics that make adolescents more or less susceptible to the effects of their peer status on their weight perception and dieting. Four such characteristics were examined: liking and disliking, self-esteem, and gender.

#### Liking and disliking

Not all popular adolescents are liked by their peers (de Bruyn & Cillessen, 2006; Rodkin et al., 2000). The associations of popularity with weight perception and dieting may depend on liking and disliking. Popular adolescents who are disliked may be distressed by their low likeability and therefore do everything they can to maintain their status (Rancourt & Prinstein, 2010). Popularity status insecurity was related to body dissatisfaction and, indirectly, a drive for thinness and restrained eating (Li & Li, 2023). In addition, both high popularity and low likeability have predicted negative weight-related cognitions and behaviors (Rancourt & Prinstein, 2010). Being liked could protect adolescents against distress over status, which could benefit healthy weight perception and eating behavior (Graham et al., 2000; Rancourt & Prinstein, 2010). Previous studies only examined liking on a continuum and as a predictor, but liking and disliking may play an important moderating role in the associations between popularity with weight perception and dieting over time.

While popular adolescents vary in likeability, unpopular adolescents typically are not liked (Rodkin et al., 2000). Given this limited variability, likeability may not moderate the associations of unpopularity with weight perceptions and dieting. However, in theory, when unpopular adolescents *are* liked, their weight perceptions may be less negative and they may diet less over time. The opposite may be the case for disliking. As there was no basis in theory or previous research for clear expectations in this matter, these moderations were examined in an exploratory manner.

#### Self-esteem

Low self-esteem is a consistent risk factor, and high self-esteem a consistent protective factor, in the development of eating pathology (e.g., Pearson et al., 2017). In a large cross-sectional study, self-esteem strongly differentiated adolescents who did and did not engage in unhealthy dieting (Croll et al., 2002). In a prospective study, low self-esteem was bi-directionally related to emotional and restrained eating within persons (Beckers et al., 2024). In adolescence, self-esteem is shaped in part by social support from peers and high self-esteem is associated with positive social support (Mann et al., 2004). Popular peers with high self-esteem may therefore feel less pressure to diet to meet group norms and expectations than popular peers with low self-esteem. Thus, self-esteem may moderate the association of social status with weight perception and dieting.

#### Gender

There may be important gender differences in the associations of popularity and unpopularity with body image. For females, pubertal changes such as widening hips and increasing weight are incongruent with the “thin ideal” for women and cause females to be more concerned about their bodies than males (McCabe et al., 2002). Males in general become more muscular and taller in puberty, which is congruent with societal ideals for men. They may be perceived more positively throughout their development for this reason (McCabe et al., 2002). Studies suggest that appearance is more important in females’ peer relations than among males (Vannatta et al., 2009). It was expected that the associations of popularity and unpopularity with weight perception and dieting over time would be larger for females than for males.

## Current Study

There are important concerns about adolescents’ weight perception and dieting, and the role of the peer group in these potentially health risk cognitions and behaviors. There is hardly any research on the associations of social status in the peer group and adolescents’ body image and the characteristics that may exacerbate or temper these associations. Popularity and unpopularity have not been studied as separate constructs in this relationship. This study had two research questions. The first question regards whether there are associations of popularity and unpopularity with adolescents’ weight perception and dieting behavior over time (Research Question 1). It was expected that popularity would predict positive weight perception over time (Hypothesis 1a), but also more dieting over time (Hypothesis 2a). It was expected that unpopularity would predict more negative weight perception over time (Hypothesis 1b) and more dieting over time (Hypothesis 2b). The second question was whether these associations were moderated by liking and disliking, self-esteem, and gender (Research Question 2). Liking was expected to increase the association of popularity with positive weight perception (Hypothesis 3a) and decrease the association of popularity with dieting (Hypothesis 4a) over time. Disliking was expected to decrease the association of popularity with positive weight perception (Hypothesis 5a) and increase the association of popularity with dieting (Hypothesis 6a) over time. The interactions of liking with unpopularity (exploratory Hypothesis 3b and Hypothesis 4b) and disliking with unpopularity (exploratory Hypothesis 5b and Hypothesis 6b) were explored. Self-esteem was expected to increase the association of popularity with positive weight perception (Hypothesis 7a) and decrease the association of popularity with dieting (Hypothesis 8a) over time. Self-esteem was expected to decrease the associations of unpopularity with negative weight perception (Hypothesis 7b) and dieting (Hypothesis 8b) over time. Finally, the (longitudinal) associations of popularity and unpopularity with weight perception (Hypothesis 9a and Hypothesis 9b) and dieting (Hypothesis 10a and Hypothesis 10b) were expected to be larger for females than males.

## Method

### Participants

Participants were part of the Kandinsky Longitudinal Study (KLS), an ongoing cross-sequential study of the social and academic development of youth in secondary education (Stoltz et al., 2016). The current study used three waves of data, collected in 2013 (T1), 2014 (T2), and 2015 (T3) at one high school in the Netherlands, with a middle class and mostly white population. The majority of the adolescents at this school are born in the Netherlands and have parents born in the Netherlands (Verheijen et al., 2020). Participants were 1697 adolescents in the first four years of secondary education (the equivalent of US Grades 7 to 10). One cohort of 478 participants (28.17%) participated in all three waves and 587 adolescents (34.59%) participated in two out of three waves. Dutch secondary schools have three main academic tracks: VWO (college preparatory), HAVO (preparation for higher administrative training), and VMBO (prepation for vocational and technical training). In the beginning grades of secondary education, tracks are sometimes combined (HAVO/VWO, VMBO/HAVO). The distribution of participants across tracks was 6.3% VMBO, 20.3% VMBO/HAVO, 20.2% HAVO, 40.0% HAVO/VWO, and 13.1% VWO. Most participants were 12-16 years old (*M* = 14.18 years, *SD* = 1.29). There were 1072 participants (501 males, 521 females) from 42 classes at T1, 1086 (528 males, 558 females) from 41 classes at T2, and 1083 (533 males, 550 females) from 40 classes at T3, yielding a total of 3241 observations, see Supplementary Table 1. The classes included 17 to 31 students and the overall participation rate across classrooms was above 90%. One participant with an extreme weight score, assumed to be a data entry mistake, was removed from the dataset. Given the large amount of supplementary materials, please consult Table 1 for an overview of what information on the methods and results is reported where.

**Table 1:** Overview of what methods and results are in the supplementary materials and what in the main section

Methods	Main section	Supplementary materials
Description participants	Method section (Participants)	
Sample size by wave and grade		Supplementary Table 1
Description of weight perception	Shortly in method section (Measures)	
Descriptive statistics and computation of weight perception		Supplementary Text 1 + Table 2 + Fig. 1
Description of dieting	Shortly in method section (Measures)	
Descriptive statistics and computation of dieting measures		Supplementary Text 1 + Table 2 + Fig. 2

Results	Main section	Supplementary materials
Descriptive statistics main variables per wave + intercorrelations		Supplementary Table 4
Descriptive statistics main variables across three waves per gender		Supplementary Table 5
Gender differences tested with *t*-tests and χ^2^ tests		Supplementary Table 5 + Supplementary Text 2
Correlations between main study variables within each timepoint		Supplementary Table 6
Description correlations between main study variables within each timepoint	Result section	
Results of the mixed effects models	Table 1 + Result section + Figs. 1 and 2	

### Procedure

The participating school formally requested the KLS project in 2010 to gain insight into students’ social and emotional problems. The school requested parental permission at the beginning of each school year for all examinations and studies, including the KLS project. Parents were informed about the purpose and procedures of the study and could exclude their child from participation. Adolescents provided consent at the beginning of each survey. This procedure was approved by the Institutional Review Board of the Faculty of Social Sciences at Radboud University (protocol number ECSW-2018-086, Stoltz et al., 2016).

Data collection took place in the classroom at school and took approximately 45 to 60 min. Before the start, participants were informed of the goal of the study and could ask questions. The instructions included clear explanations of confidentiality and anonymity, and of the ethical issues regarding the computerized survey (e.g., explanation of storage of code numbers instead of names).

Adolescents sat at a private desk, with a divider around their computer screen so that it could not be seen by other students. There were always two researchers in the classroom to give instructions and answer questions. The computerized survey started with an informative introduction. Students had to read the instruction and consent by clicking “start” before they could proceed. The computerized survey included peer nominations and self-report questions of weight perception, dieting, and self-esteem. Other measures in the survey were beyond the scope of this study.

The reference group for the peer nominations was the classroom. Participants could name as many or as few classmates for each question as they wished, but not themselves. Each nomination item was shown at the top of a new screen on the computer, with the list of potential nominees below the question. Participants could nominate a peer (e.g., as popular or liked) by clicking on their name, which then turned from black to gray. This further eliminated the possibility that students could see each other’s nominations (Stoltz et al., 2016). The order of names was randomized between participants, but stayed the same for all questions within participants. A procedure was built in requiring participants to provide at least one nomination for each question. This was done to prevent that participants would randomly click through the screens. Nevertheless, participants were never forced to answer a question. Following the ethics guidelines for sociometric studies, they were explicitly told that they could stop at any moment, with no negative consequences (Guideline 2, Bell‐Dolan & Wessler, 1994). If a student indicated that they did not know whom to nominate for a question, one of the researchers clarified the question to the student and emphasized again that all answers were stored anonymously (Stoltz et al., 2016).

### Measures

#### Weight perception

Weight perception was measured with one item (‘How would you describe your weight?’; World Health Organization, 2015). This item had five answer categories: (1) I am extremely underweight, (2) I am underweight, (3) I am just about the right weight, (4) I am overweight, and (5) I am extremely overweight. This item was transformed into a more easily interpretable continuous weight perception variable by recoding 1 and 2 (feeling underweight) into negative scores (−2 and −1), 3 (feeling the right weight) into 0, and 4 and 5 (feeling overweight) into positive scores (1 and 2). The main interest was in whether adolescents perceived their weight as right or not, that is, whether they felt positively or negatively about their weight, regardless of whether they perceived themselves as underweight or overweight (as the source of a negative weight perception). Therefore, a binary weight perception variable was created from the recoded continuous variable by combining feeling underweight (−1 and −2) and feeling overweight (1 and 2) into one new score (1), indicating being negative about one’s weight, and leaving 0 as it was, indicating being positive about one’s weight. For a detailed report of the descriptive statistics and computation of this variable, see the Supplementary Text 1.

#### Dieting behavior

Dieting was assessed with two items. The first question was: “In the past 12 months, how many times have you followed a diet in an attempt to lose or maintain your weight?”. This question had 5 answer categories: (0) never, (1) 1-2 times, (2) 3-4 times, (3) 5-6 times, and (4) 7 times or more. Because this variable was positively skewed, a binary variable was computed indicating whether a participant had dieted (scored 1) or not (scored 0) in the past year. This binary variable for dieting was a dependent variable in the main analyses and is from now on referred to as “*dieting*”. Supplementary Table 2 provides the descriptive statistics per wave. A second question about dieting*, “current weight control”*, was used for exploratory purposes. For a detailed report of the descriptive statistics of these two variables, see the Supplementary Text 1.

#### Popularity and likeability

The scores for popularity, unpopularity, liking and disliking were derived from the sociometric and peer assessment in the classroom (see Procedure). The scores were derived from four peer nominations: popularity (‘Who are most popular?’), unpopularity (‘Who are least popular?’), liking (‘Who do you like the most?’) and disliking (‘Who do you like least?’). These nominations yield reliable and valid measures of social status in classrooms (see, e.g., van den Berg & Cillessen, 2013). Nominations received for each item were counted and divided by the number of nominating peers to create proportion scores. A score of 1.0 would indicate that an adolescent was named by all participating classmates for a particular question.

#### Self-esteem

Self-esteem was measured with the 10-item Dutch version of the Rosenberg Self-Esteem Scale (Rosenberg, 1965). An example item is: “On the whole, I am satisfied with myself”. All items were rated on a 4-point Likert scale (1 = strongly disagree, 4 = strongly agree). The score for self-esteem was created by taking the average of the 10 ratings, with higher scores indicating more self-esteem. Cronbach’s α across the 10 items was 0.88, 0.88, and 0.89 at T1, T2, and T3, respectively.

### Data Analyses

The data were restructured to long format, with time points nested within participants. The analyses were performed in R (R Core Team, 2023). Four series of five linear mixed-effects models were run using the *lme4* package (Bates et al., 2015). All available data were used in the mixed-effects (i.e., multilevel) models, as is done by default in the lme4 package in R. Time was centered at T1 and coded as 0, 1, and 2 for T1 to T3, respectively, given equal 1-year intervals between the consecutive waves. As the dependent variables were binary, the family was specified as binomial.

The dependent variables weight perception and dieting were time varying; the independent variables (popularity, unpopularity), covariate (gender) and moderators (liking, disliking, self-esteem and again gender) were time-invariant and taken from T1. To test the first research question, the main effects of popularity and unpopularity were tested, with gender as a covariate. The test the second research question, the effects of the moderators were tested, again with gender as a covariate. All models were run twice: once for weight perception and once for dieting as the dependent variable.

In the first model of each series, the effects of time, popularity, and their interaction were the predictors. Gender was included as a covariate. The effect of time indicated changes in the dependent variable across the three years of the study. Gender, time, popularity, and the time × popularity interaction were added as fixed effects. In three subsequent models, each of the moderators liking, disliking and self-esteem was tested separately. This involved adding the moderator as a main effect, the interaction of the moderator with popularity, and the interactions of both terms with time. In the fifth model, in which gender was the moderator, the time × gender, gender × popularity, and time × gender × popularity interactions were added to the first model. These five models were then repeated with unpopularity as the predictor (unpopularity, unpopularity+liking, unpopularity+disliking, unpopularity+self-esteem, and unpopularity+gender). These 10 models in total were run once predicting weight perception and once predicting dieting, yielding a total of 20 models.

All continuous predictors were centered, and gender was sum-to-zero coded (males = −1 and females = 1). A random intercept varying over participants was included. The intraclass correlation of classroom was very low (<0.05); therefore nesting in classrooms was not accounted for. Models including a random slope for time were run, varying over participants. Because some of these models were unidentifiable and most of them produced convergence and/or singularity warnings which could not be solved, the models were simplified by excluding this random slope. The exact model specifications can be found in our R script (https://osf.io/62wvx).

Following the advice of Barr et al. (2013) for generalized mixed-models, *p*-values were determined using Type 3 likelihood ratio test (LRT), using the mixed() function from the package afex (Singmann et al., 2023). Four different types of models were run defined by four predictor-outcome combinations: popularity-weight perception, unpopularity-weight perception, popularity-dieting, and unpopularity-dieting. Therefore, *p* = 0.05 was divided by four and the criterion for significance was set to *p* = 0.0125. Significant interaction effects were followed up by post-hoc analyses. For the moderators liking, disliking, and self-esteem, these consisted of simple slope analyses at low (*M* − 1 *SD*) and high (*M* + 1 *SD*) levels of the moderator. For gender as the moderator, the post-hoc tests used females fixed as 1 and males fixed as 0 to interpret an effect for females, and vice versa to interpret an effect for males (see Sommet & Morselli, 2017).

## Results

### Descriptive Statistics

Descriptive statistics of the main variables in each wave were computed including all observations per wave, using the packages Pastecs (Grosjean & Ibanez, 2018) and Lattice (Sarkar, 2008) and are shown in Supplementary Table 4. The descriptive statistics were also examined by gender, using all observations across the three waves. Because gender differences were not the main focus of this study, descriptive statistics, *t*-tests and χ^2^ tests by gender are in the Supplementary Materials (see Supplementary Text 2 and Table 5).

### Correlations between Main Study Variables

Supplementary Table 4 shows the stability of the variables, computed as Pearson’s correlations over time using the Hmisc package (Harrel, 2023). Correlations were also computed between the main variables within each time point (see Supplementary Table 6). At T1, liking was significantly negatively associated with negative weight perception. Thus, a higher level of liking was associated with more positive weight perception. Self-esteem was negatively associated with negative weight perception and dieting. Thus, as expected, self-esteem was associated with more positive weight perceptions and less dieting. Negative weight perception and dieting were significantly positively associated. At T2, the pattern of correlations was similar to T1, except that liking was not significantly associated with weight perception. At T3, popularity was significantly positively related to self-esteem and significantly negatively to negative weight perception. Thus, more popular adolescents had higher self-esteem and more positive weight perception. Unpopularity was negatively related to self-esteem and positively to negative weight perception. Thus, more unpopular adolescents had lower self-esteem and more negative weight perception.

### Mixed-Effects Models

For the main analyses, the glmer function of the lme4 package (version 1.1.20; Bates et al., 2015) was used in R (R Core Team, 2023). In 15 of the 20 models, convergence and singularity warnings occurred. Switching to the optimizer bobyqa removed these warnings. None of the models yielded a significant effect of time on weight perception or dieting (see Table 2), indicating no general increase or decrease in weight perception and dieting over the study period. In other words, on average, the means of weight perception and dieting remained constant across the study waves.

**Table 2:** Results of mixed-effects models for the prediction of weight perception and dieting

	Negative weight perception				Dieting			
	Estimate	*SE*	OR	*p*	Estimate	*SE*	OR	*p*
**RQ 1**								
Popularity								
Time	−0.06	0.10	0.94	0.546	0.07	0.09	1.07	0.415
Popularity	−0.64	0.61	0.53	0.292	0.49	0.50	1.63	0.334
Time × Popularity	−0.45	0.43	0.64	0.296	0.02	0.39	1.02	0.950
Gender	0.40*	0.14	1.49*	0.002	1.25*	0.13	3.49*	<0.001
Unpopularity								
Time	−0.06	0.10	0.94	0.522	0.07	0.09	1.07	0.441
Unpopularity	1.17	0.66	3.22	0.070	0.77	0.50	2.16	0.126
Time × Unpopularity	0.36	0.40	1.43	0.373	−0.07	0.36	0.93	0.849
Gender	0.42*	0.14	1.52*	0.002	1.24*	0.13	3.46*	<0.001
**RQ 2**								
Popularity & liking								
Time	−0.04	0.10	0.96	0.728	0.06	0.09	1.06	0.516
Popularity	−0.79	0.69	0.45	0.251	0.71	0.56	2.03	0.203
Time × Popularity	−0.21	0.47	0.81	0.651	−0.03	0.41	0.97	0.943
Gender	0.49*	0.14	1.63*	< 0.001	1.30*	0.13	3.67*	<0.001
Liking	−5.31*	1.60	<0.01*	0.001	−3.20*	1.27	0.04*	0.010
Time × Liking	0.179	1.15	1.20	0.876	0.68	1.02	1.97	0.505
Popularity × Liking	19.78*	6.19	**3.89 ×10**^8*^	0.001	4.47	4.83	87.36	0.356
Popularity × Liking × Time	−5.78	4.13	<0.01	0.157	0.09	3.68	1.09	0.981
Popularity & disliking								
Time	−0.06	0.10	0.94	0.523	0.07	0.09	1.07	0.445
Popularity	−0.60	0.62	0.55	0.338	0.58	0.51	1.79	0.256
Time × Popularity	−0.45	0.43	0.64	0.296	<−0.01	0.39	1.00	0.996
Gender	0.42*	0.14	1.52*	0.002	1.27*	0.13	3.56*	<0.001
Disliking	2.32	1.31	10.18	0.070	2.19*	1.02	8.94*	0.032
Time × Disliking	−1.04	0.87	0.35	0.224	0.40	0.75	1.49	0.593
Popularity × Disliking	−5.63	5.12	< 0.01	0.270	−7.46	4.25	<0.01	0.076
Popularity × Disliking × Time	1.28	3.68	3.60	0.730	1.90	3.15	6.69	0.547
Popularity & self-esteem								
Time	0.02	0.10	1.02	0.848	0.09	0.10	1.09	0.373
Popularity	−0.66	0.65	0.52	0.308	0.41	0.51	1.51	0.423
Time × Popularity	−0.64	0.47	0.53	0.168	−0.13	0.41	0.88	0.756
Gender	−0.08	0.15	0.92	0.572	0.99*	0.13	2.69*	<0.001
Self-esteem	−2.29*	0.36	0.10*	<0.001	−1.48*	0.25	0.23*	<0.001
Time × Self-esteem	0.02	0.19	1.02	0.934	−0.08	0.17	0.92	0.654
Popularity × Self-esteem	−0.51	1.11	0.60	0.647	0.78	0.85	2.18	0.367
Popularity × Self-esteem × Time	−0.26	0.76	0.77	0.733	0.26	0.64	1.30	0.687
Popularity & gender								
Time	−0.05	0.10	0.95	0.621	0.05	0.09	1.05	0.607
Popularity	−0.57	0.61	0.57	0.349	0.40	0.52	1.49	0.448
Time × Popularity	−0.44	0.44	0.64	0.308	0.10	0.40	1.11	0.798
Gender	0.44*	0.15	1.55*	0.002	1.18*	0.14	3.25*	<0.001
Time × Gender	−0.06	0.10	0.94	0.566	0.11	0.09	1.12	0.237
Popularity × Gender	0.42	0.61	1.52	0.487	1.39*	0.52	4.01*	0.006
Popularity × Gender × Time	0.56	0.44	1.75	0.195	−0.28	0.40	0.76	0.490
Unpopularity & liking								
Time	−0.02	0.11	0.98	0.832	0.07	0.10	1.07	0.515
Unpopularity	0.40	0.91	1.49	0.661	0.34	0.73	1.40	0.638
Time × Unpopularity	0.85	0.62	2.34	0.169	0.08	0.57	1.08	0.893
Gender	0.48*	0.14	1.62*	<0.001	1.27*	0.13	3.56*	<0.001
Liking	−4.53*	1.76	0.01*	0.009	−2.21	1.42	0.11	0.118
Time × Liking	0.57	1.24	1.77	0.649	0.83	1.13	2.29	0.462
Unpopularity × Liking	1.45	8.60	4.26	0.867	−0.71	6.77	0.49	0.917
Unpopularity × Liking × Time	5.98	6.05	395.44	0.322	0.06	5.31	1.06	0.991
Unpopularity & disliking								
Time	−0.06	0.10	0.94	0.577	0.07	0.09	1.07	0.444
Unpopularity	1.03	0.75	2.80	0.162	0.30	0.57	1.35	0.607
Time × Unpopularity	0.69	0.47	1.99	0.134	−0.13	0.42	0.88	0.749
Gender	0.43*	0.14	1.54*	0.001	1.27*	0.13	3.56*	<0.001
Disliking	2.07	1.49	7.92	0.162	1.93	1.21	6.89	0.110
Time × Disliking	−1.41	1.00	0.24	0.153	0.47	0.89	1.60	0.600
Unpopularity × Disliking	−2.73	3.96	0.07	0.493	1.13	3.08	3.10	0.713
Unpopularity × Disliking × Time	−1.13	2.77	0.32	0.684	−0.12	2.29	0.87	0.957
Unpopularity & self-esteem								
Time	0.02	0.11	1.02	0.830	0.08	0.10	1.08	0.395
Unpopularity	0.82	0.71	2.27	0.245	0.58	0.51	1.79	0.261
Time × Unpopularity	0.54	0.45	1.72	0.224	0.07	0.38	1.07	0.865
Gender	−0.05	0.15	0.95	0.725	0.96*	0.12	2.61*	<0.001
Self-esteem	−2.32*	0.37	0.10*	<0.001	−1.44*	0.25	0.24*	<0.001
Time × Self-esteem	<0.01	0.19	1.00	0.995	−0.07	0.17	0.93	0.674
Unpopularity × Self-esteem	−1.38	1.28	0.25	0.272	0.94	0.91	2.56	0.301
Unpopularity × Self-esteem × Time	1.05	0.79	2.86	0.181	−0.06	0.68	0.94	0.933
Unpopularity & gender								
Time	−0.06	0.10	0.94	0.555	0.05	0.09	1.05	0.610
Unpopularity	1.15	0.68	3.16	0.082	0.89	0.51	2.44	0.079
Time × Unpopularity	0.40	0.41	1.49	0.331	−0.16	0.37	0.85	0.672
Gender	0.47*	0.16	1.60*	0.001	1.17*	0.14	3.22*	<0.001
Time × Gender	−0.06	0.10	0.94	0.554	0.11	0.09	1.12	0.242
Unpopularity × Gender	1.12	0.68	3.06	0.089	−0.80	0.50	0.45	0.114
Unpopularity × Gender × Time	−0.68	0.41	0.51	0.098	0.44	0.37	1.55	0.242

Estimates represent the log-odds. 15 models were estimated with optimizer Bobyqa to avoid convergence and singularity warnings. *n* = 957 in models with self-esteem, *n* = 1047 in all other models. Computed *p*-values using type 3 LRT

Statistically significant effects (corrected *p*-value < 0.0125) are denoted in bold

*p < 0.05 (uncorrected)

#### Research question 1

Popularity and unpopularity at T1 did not have a significant main effect on weight perception, and the interactions of time with popularity and unpopularity were not significant (see Table 2). That is, popularity and unpopularity did not predict weight perception at T1 or *changes* in weight perception from T1 to T3. H1a and H1b were not confirmed. Popularity and unpopularity at T1 also did not have a significant main effect on dieting, and the interactions of time with popularity and unpopularity were not significant (see Table 2). Thus, popularity and unpopularity did not predict dieting at T1 or *changes* in dieting from T1 to T3. H2a and H2b were not confirmed.

#### Research question 2

The three-way interaction of time, popularity or unpopularity, and a moderator was not significant for any of the moderators. Thus, the four moderators did not affect the associations of popularity and unpopularity with changes in weight perception and dieting over time. Below the interactions without time are presented, indicating interaction effects on weight perception and dieting at T1 (not on *changes* from T1 to T3).

##### Moderation of the effects of popularity on weight perception

**Fig. 1:**
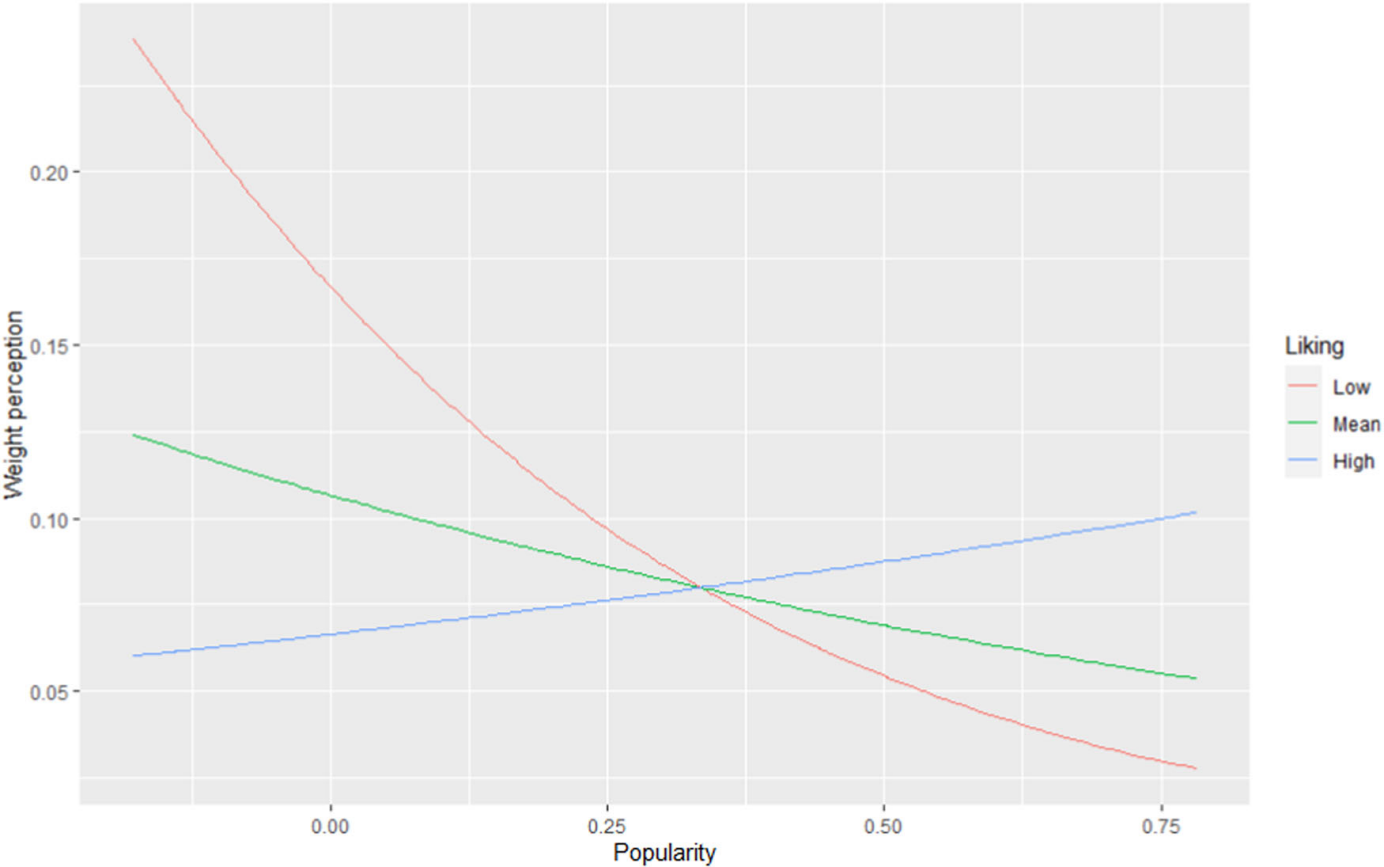
Predicted probabilities of negative weight perception (Y-axis): interaction between popularity (X-axis) and liking (different levels represented by the different lines)

Liking significantly moderated the association between popularity and weight perception at T1 (see Table 2). When liking was high, popularity predicted more negative weight perception than when liking was low (see Fig. 1). To interpret this interaction, post-hoc analyses with two dummy-coded models were conducted. Simple slope analysis showed that at high levels of liking (*M* + 1 *SD*), the association between popularity and negative weight perception was not significant (*b* = 1.16, *SE* = 0.74, *OR* = 3.18, *p* = 0.119). At low levels of liking (*M*-1*SD*), the association between popularity and a negative weight perception was significant and negative; a 1-unit increase in popularity results in 0.06 times more chance (or rather 16.67 less chance) of a negative weight perception (*b* = −2.74, *SE* = 1.08, *OR* = 0.06, *p* = 0.011). Thus, the association between popularity and negative weight perception was clearly negative when liking was low, but not significant when liking was high. This was in contrast to H3, which stated that more liking would increase the association between popularity and positive weight perception. The other moderators (disliking, self-esteem and gender) did not significantly interact with popularity, thus hypotheses 5, 7a, and 9a were not confirmed.

The main effects of the moderators liking, self-esteem, and gender on weight perception were significant (see Table 2). Liking was negatively associated with a negative weight perception, the odds of having a positive weight perception were higher when liking at T1 was higher. There was no significant main effect of disliking. Self-esteem was negatively associated with a negative weight perception; the odds of a positive weight perception were higher when self-esteem at T1 was higher. Gender had a significant main effect on weight perception; the odds of having a more negative weight perception were higher for females than for males.

##### Moderation of the effects of unpopularity on weight perception

It was explored whether there was an interaction of liking and disliking with unpopularity, which was not the case (see Table 2). Self-esteem and gender did not significantly interact with unpopularity in the prediction of weight perception. Thus, hypotheses 7b and 9b were not confirmed.

Examining the main effects of the moderators, liking, self-esteem and gender significantly predicted weight perception. Liking was negatively associated with a negative weight perception, the odds of a positive weight perception were higher when liking at T1 was higher. Disliking did not predict weight perception. Self-esteem was negatively associated with a negative weight perception, the odds of a positive weight perception were higher when self-esteem at T1 was higher. Gender (which was a covariate in all models) had a significant main effect on weight perception in all models with unpopularity, except for the model with self-esteem as the moderator (see Table 2). The odds of a more negative weight perception were higher for females than for males.

#### Moderation of the effects of popularity on dieting

**Fig. 2:**
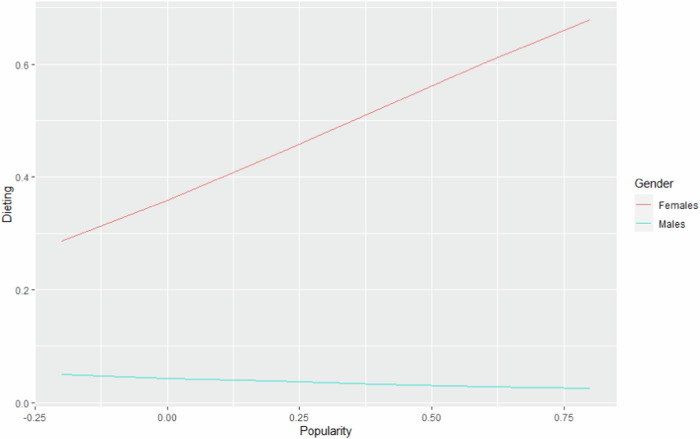
Predicted probabilities of dieting (Y-axis): the interaction between popularity (X-axis) and gender (represented by the different lines)

Liking, disliking and self-esteem did not significantly interact with popularity in the prediction of dieting (see Table 2). Thus, hypotheses 4, 6 and 8a were not confirmed. Gender significantly moderated the association between popularity and dieting (see Fig. 2). To interpret the interaction, post-hoc analyses with two dummy-coded models were conducted. Simple slope analysis showed that for males, the association between popularity and dieting was not significant (*b* = −1.00, *SE* = 0.76, *OR* = 0.37, *p* = 0.190). For females, the association between popularity and dieting was significantly positive, with each 1-unit increase in popularity the odds of having dieted in the past year were multiplied by 5.98 (*b* = 1.79, *SE* = 0.71, *OR* = 5.98, *p* = 0.012). These analyses showed that popularity predicted more dieting for females, but not for males. H10a was confirmed regarding the concurrent association.

Examining the main effects, liking, self-esteem and gender were significantly related to dieting. Disliking (after correcting the *p*-value for multiple testing) was not. Liking and self-esteem were negatively associated with dieting, the odds of having dieted in the past year were lower at higher levels of liking and self-esteem. For gender, the odds of having dieted in the past year were higher for females than for males.

##### Moderation of the effects of unpopularity on dieting

The interactions of liking and disliking with unpopularity were explored, which were not significant (see Table 2). Self-esteem and gender did not significantly interact with unpopularity in the prediction of dieting. Hypotheses 8b and 10b were not confirmed.

Liking and disliking did not have significant main effects on dieting; self-esteem and gender did. Self-esteem was negatively associated with dieting. The odds of having followed a diet in the past year were lower when self-esteem at T1 was higher. The odds of having followed a diet in the past year were higher for females than for males.

### Sensitivity Analyses

As sensitivity analyses, zBMI was added as a covariate to all models. This did not change the pattern of results in any of the models. ZBMI itself had a significant positive association with negative weight perception and dieting in all models. In addition, it was checked whether the results were the same when gender was not included as covariate. All models showed the same pattern of results, except for the model with unpopularity as the predictor, liking as the moderator, and weight perception as the outcome, and the model with popularity as the predictor, liking as the moderator, and dieting as the outcome. In these models, the main effect of liking on weight perception (*p* = 0.036) and on dieting (*p* = 0.623) was no longer significant (after correcting the *p*-value for multiple testing). This indicates that it was important to include gender as a covariate in the models.

## Discussion

There is a scarcity of research on the (longitudinal) relationship between (un)popularity and body image. The existing literature on this topic does not distinguish popularity from unpopularity, nor liking from disliking, while unique effects can be expected. Knowledge is needed of the characteristics that make adolescents susceptible to the effects of (un)popularity on weight perception and dieting. The current study used a longitudinal sample of early adolescents and mixed-effects models to examine the associations of popularity and unpopularity with weight perception and dieting over time and possible moderators (liking, disliking, self-esteem, and gender) of these associations. There were no longitudinal associations between (un)popularity and weight perception or dieting, but there were two significant moderator effects and several significant main effects in the concurrent associations.

### (Un)popularity and Weight Perception

The mixed-effects models showed that popularity did not predict positive weight perception, and unpopularity did not predict negative weight perception concurrently or over time. This is not in line with the hypotheses, nor with previous studies in which popularity was related to positive weight perception (Borch et al., 2011; Rancourt & Prinstein, 2010) and low popularity to negative weight perception (Gorman et al., 2011; Wang et al., 2006). The results suggest that the effect of peer status on weight perception and dieting applies primarily to adolesents in general. It may be that other social factors today explain much more of these weight-related constructs, such as the influence of social media. Social media lower the threshold to (upward) social comparisons, as individuals portray themselves as best as they can on social media platforms (Chou & Edge, 2012) and content can be viewed 24/7. Features of social media use such as viewing appearance-focused content and negative online social comparisons are known to increase body dissatisfaction (Dane & Bhatia, 2023). Viewing peers online goes beyond the classroom, which could explain why peer status in the classroom may have a more limited role in weight-related constructs than previously expected.

The fact that there were no associations between popularity and weight perception in general, does not mean they do not exist in subgroups. The findings unexpectedly showed that when liking was low, high popularity was related to a more positive weight perception concurrently. In adolescents low in liking, popularity *was* associated with more positive weight perception. This finding was in contrast with the hypotheses, which stated that popular adolescents high in liking would have more positive weight perceptions than those low in liking. Adolescents low in liking and high in popularity may not be distressed by their low liking (as previously reasoned) as long as they maintain their popular status (LaFontana & Cillessen, 2010; van den Broek et al., 2016). Research has shown that the combination of high popularity and low likeability is associated with bullying (e.g., de Bruyn et al., 2010) and adolescents who engage in bullying find being popular more important than being liked (Garandeau & Lansu, 2019). Second, adolescents high in popularity and low in liking may not need to diet to attain a body considered to be ideal, because they may already have it, which may have helped them to become popular in the first place. Borch et al. (2011) found that attractiveness and aggression together predicted more popularity, and less likeability. This suggests that adolescents high in popularity and low in liking are attractive, and may have a lower need to diet.

This study builds upon the study by Rancourt and Prinstein (2010), who found that high popularity and low likeabilty separately predicted negative weight-related behaviors. The current study examined the interaction of popularity and liking; when likeability was low, high popularity predicted positive weight perception. Apparently, there is something unique about being popular but not liked. This suggest that there is variability in the group of popular adolescents, where only those low in liking have more positive weight perceptions. This fits with previous literature on the heterogeneity of popularity (e.g., Parkhurst & Hopmeyer, 1998; Rodkin et al., 2000), which consistently distinguishes a popular-liked and a popular-disliked group with different characteristics and outcomes. It is interesting that there was only an effect for adolescents low in liking, but not high in disliking. Because Rancourt and Prinstein (2010) considered popularity and likeability as single continuum, the results are not directly comparable. Further research is needed to better understand how and why different combinations of social status relate to body image constructs.

The result that unpopularity was not related to negative weight perception is very unexpected, as in previous research unpopularity was associated with less ‘ideal’ body shapes (Wang et al., 2006) and victimization (Gorman et al., 2011), and appearance victimization decreased body satisfaction (Webb & Zimmer-Gembeck, 2014). Perhaps adolescents with a less ‘ideal’ appearance tend to be unpopular, but this may not necessarily be related to their weight perception as some unpopular adolescents do not care much about group norms (Schwartz & Gorman, 2011). They may worry less about not having the ideal body. Thus, unpopularity in itself was not a risk factor for negative weight perception, which provides a positive message about unpopular adolescents.

### (Un)popularity and Dieting

The hypothesis that popularity was related to dieting was not confirmed, neither concurrently nor longitudinally. It seems that dieting is not a health risk behavior predicted by popularity, while popularity does predict other health risk behaviors (Cillessen et al., 2023). It was reasoned that if popular adolescents focus more than other youths on adhering to social norms (Cillessen et al., 2011; Valente et al., 2005), they would diet more to attain the “ideal body”. However, it could be that dieting is simply not necessary for popular adolescents, if they already have a body that is regarded ideal (Wang et al., 2006).

The mixed model analyses did show that higher popularity was associated concurrently with a higher chance of dieting in females, but not in males. The overall association between popularity and dieting may have not been found because it is only there for females. As said before, appearance is more important in females’ peer relations than among males (Vannatta et al., 2009). For females, dieting may be a way to maintain status, as being thin is an appearance goal for females (McCabe et al., 2002). As appearance is less important for status in males, dieting would be less important to them. Additionally, dieting is mainly associated with losing weight, while males sometimes want to increase weight and become more muscular (McCabe et al., 2002). Future studies should include other cognitions (e.g., perception of muscularity) and behaviors (e.g., sports) that may be especially relevant for males.

Unpopularity was not related to dieting. This could be explained in several ways. First, as indicated, adolescents who fail to conform to group norms and behaviors are likely to be unpopular (Schwartz & Gorman, 2011). Unpopular adolescents may not only fail to conform to group norms (such as related to appearance), they may also be less willing to behave according to group norms (i.e., not willing to diet to change their weight). Future research should examine this hypothesis. Second, the effects of unpopularity on dieting may not be found immediately in adolescence, but may appear later in life. For example, Smink et al. (2018) found that unpopularity predicted eating pathology at a later age. This hypothesis could be tested with longitudinal data of late adolescents or emerging adults.

### Main Effects of Liking, Self-esteem and Gender

The analyses yielded other interesting findings about adolescents’ weight perception and dieting. One is that being liked and self-esteem predicted positive weight perception and a lower chance of dieting concurrently. This is in line with the literature on eating disorders and body image (e.g., Pearson et al., 2017, Rancourt & Prinstein, 2010). This implies that it is important to create a positive classroom climate where adolescents feel comfortable and liked.

Females had more negative weight perception and dieted more than males and as discussed before, for females, popularity predicted more dieting. This is in line with popular media that often suggest that females in particular are at risk for eating disorders, and with other studies finding lower body satisfaction for females than males (e.g., Gualdi-Russo et al., 2022). It is very important to take gender differences into account when studying this topic further. As mentioned before, it is also important to examine whether other appearance-focused cognitions and behaviors are more important or problematic for males.

### Strengths, Limitations and Suggestions for Further Research

The four nominations (most popular, least popular, liked most, liked least) were taken as separate constructs. In sociometric research, it is common to create composite scores (most popular minus least popular; liked most minus liked least; Stoltz et al., 2016). A disadvantage of considering the constructs separately, is that this makes this study more difficult to compare to previous literature. Future research should take the constructs separately again, so that a strong empirical basis can be created for the unique effects they can have. Treating the constructs separately namely allows for a broader understanding of peer subgroups and their attributes (Gorman et al., 2011). Unique results for each construct were found (while in some cases, popularity and liking were associated with weight perception and dieting, unpopularity and disliking were not), which underlines the importance of considering them separately. Previous studies suggest that as well. For example, whereas popularity primarily predicted externalizing risk behaviors (e.g., Mayeux et al., 2008), unpopularity primarily predicted internalizing problems such as loneliness (Prinstein & La Greca, 2002). As externalizing and internalizing behaviors may be differentially related to weight perception and dieting, it is worthwhile to continue to consider popularity and unpopularity separately in this domain. It will be very interesting to examine the associations of externalizing and internalizing behaviors themselves with weight perception and dieting, as their involvement may explain much about the links between peer status and weight-related constructs. It will matter greatly whether an adolescent diets to approach a popular thin ideal or because of a negative weight perception caused by feelings of loneliness and low self-esteem. Understanding adolescents’ underlying reasons for why some have more negative weight perceptions and diet more, is an important direction for further research.

This research had limitations regarding the dependent variables. The dependent variables were derived from one item each, which may make them less reliable, despite their high face validity. As the weight perception variable only assessed perceptions of being overweight, underweight, or about the right weight, it did not necessarily reflect positivity or negativity. Future studies could ask participants to reflect more specifically on their weight satisfaction. Additionally, taking underweight and overweight together limits the interpretation of the results. There were fewer participants in these categories than participants in the ‘right weight’ category. This made it statistically necessary to take them together. It was outside of the scope of this paper to examine differences between perception of being underweight versus overweight. Future research could take them separately and combine it with information on actual weight, which could additionally provide information on the difference between feelings about weight and actual weight.

This study included adolescents in the Netherlands, who have a Westernized body and beauty ideal. The findings may not generalize to other countries and cultures, that have different body ideals and with it different risks to adolescents’ health behavior. It will be interesting to examine the impact of cultural differences on adolescents’ body ideals. Furthermore, the statistical modeling may suggest that popularity is seen as a cause of risky health behaviors. However, as indicated by Schwartz and Gorman (2011), popularity can be a “marker” of underlying attributes such as a need for status and attention, and these attributes may determine the later negative outcomes. Adolescents who value being popular or liked may be most susceptible to peer pressure and conformity. Peer pressure and the belief that weight is important for being liked, predict dieting and low body esteem (Lieberman et al., 2001). Popularity status insecurity is related to body dissatisfaction and, indirectly, a drive for thinness and restrained eating (Li & Li, 2023). Future research should examine whether these underlying characteristics are responsible for the associations between popularity and body image outcomes. Lastly, follow-up studies could take a longer-term developmental perspective and systematically test associations in different developmental periods, for example to examine how the development of weight perception and dieting across age and grades depends on peer status and related predictors.

### Implications

This study contributed to knowledge of weight perception and dieting by zooming in on the role of social status in adolescence. There were no significant changes in weight perception and dieting over time, and no constructs in this study predicted changes in weight perception and dieting over time. Changes (as a result of social status) may occur after a delay, later in life, after adolescence. In some cases, when liking is low and for females, popularity was associated with weight perception and dieting. It is important to take gender and likeability into account when studying peer status and body image. While peer status may not always predict weight-related constructs, there is something unique about both being high in popularity and low in liking. Popularity predicted dieting in females, which can pose a risk for eating disorders later in life (Allen et al., 2014). Females may feel pressured to obtain a certain body shape for the sake of becoming more popular, which can put their health at risk. In our study unpopularity never predicted the weight-related constructs. Unpopularity does not seem to make adolescents susceptible to negative weight perception and (unhealthy) eating practices as previously expected. This provides a positive message about unpopular adolescents: They do not adhere to group norms, but this does not seem to put their body image at risk. Liking seems to have positive implications, as it generally relates to positive weight perception and less dieting, although it predicted negative weight perception in interaction with popularity. Disliking does not seem to be a risk factor for negative weight perception and dieting, which is positive. As expected, self-esteem has positive implications for weight-related constructs. Creating a positive climate in the classroom may help adolescents to be more accepting of peers who do not follow group norms or meet ideals. This will also be good for adolescents’ self-esteem, which in turn will have a positive effect on their body image. Lastly, parents and teachers should pay attention to fostering adolescents’ self-esteem, because that will enhance their body image.

## Conclusion

There are important concerns about adolescents’ body image and dieting practices. Given little previous research on the role of social status, the current study examined (1) the unique associations of popularity and unpopularity with weight perception and dieting concurrently and longitudinally, and (2) liking, disliking, self-esteem, and gender as moderators of these associations. Complex mixed-effects models were used in a longitudinal adolescent sample. Adolescents’ average weight perception and dieting were remarkably constant across the three years of the study. Popularity and unpopularity did not predict changes in weight perception and dieting over time, but there were concurrent associations. Popularity predicted positive weight perception when liking was low, and popularity predicted dieting in females. Females seem more susceptible to negative weight perception and dieting, especially in combination with concerns about their social status. For males, other appearance-related cognitions and behaviors may be more relevant. Being liked by peers and having high self-esteem seem to buffer against negative weight perception and dieting. Fostering self-esteem and positive peer relationships at school are important for adolescent health. Disliking and unpopularity were not risk factors for dieting or a negative weight perception. Future studies should examine whether peer status in adolescence predicts changes in weight-related constructs after adolescence, as problematic eating may develop later in life. This study highlighted the importance of subgroups when examining the effects of social status on body image constructs in (early) adolescents, as unique associations were found.

## Supplementary information

Supplementary Information
