# Supplementary material for: Longitudinal Associations of (Un)popularity with Weight Perceptions and Dieting in Adolescence
Source: J Youth Adolesc. 2024 Sep 25;54(3):704–19. doi: 10.1007/s10964-024-02090-8 (PMC11846728; doi:10.1007/s10964-024-02090-8)
Supplement: Supplementary file 1 — Supplementary Information [file 10964_2024_2090_MOESM1_ESM.docx]

**Supplementary Materials**

Longitudinal Associations of (Un)popularity with Weight Perceptions and Dieting in Adolescence

Aafke Swinkels*, Nina van den Broek, & Antonius H. N. Cillessen

Radboud University

Behavioural Science Institute, Radboud University, P.O. Box 9104, 6500 HE Nijmegen, The Netherlands.

*Corresponding author: Aafke Swinkels. E-mail address: aafke.swinkels@ru.nl

**Supplementary Text 1. Methods**

**Participants**

See Supplementary Table 1 for the sample sizes by wave and grade.

**Weight Perception**

The distribution of answers for the original variable Weight Perception was examined for all observations across waves, including several observations per participant (of those who have participated in more than one wave). The distribution of answers across the five answer options was severely leptokurtic. The majority of adolescents reported having about the right weight (see Supplementary Figure 1). Therefore, two new variables were created, one with three and one with two answer categories. The distributions of these variables were again examined with all observations across waves. They had better distributions than the original variable, which is shown in Supplementary Figure 1. First, a new variable with three categories was created by combining feeling underweight (scores -2 and -1) into one score (-1), feeling overweight (scores 1 and 2) into one score (+1), and leaving 0 as it was. Second, a binary variable was created by combining feeling underweight and overweight into one score (1), interpreted as being negative about one’s weight, and leaving 0 as it was, interpreted as being positive about one’s weight. This binary variable was used in the main analyses. Supplementary Table 2 provides the descriptive statistics per wave.

**Dieting**

The distribution of answers of the original variable of dieting was examined with all observations across waves. The variable was very positively skewed, with the majority of adolescents reporting never to have dieted (see Supplementary Figure 2). Therefore, the binary variable of dieting was computed (see Supplementary Figure 2). Supplementary Table 2 provides the descriptive statistics per wave.

The additional question regarding dieting was: “Are you trying to do something about your weight right now?” (referred to as “*current* *weight control*”). This question had four answer categories: (0) no, (1) yes, I am trying to lose weight, (2) yes, I am trying to gain weight, (3) yes, I am trying to maintain the same weight. As the main question of this study regarded whether adolescents diet (and how this can be predicted by other variables), and not so much about their precise goals for dieting (losing, maintaining or gaining weight), this variable was only used in a descriptive fashion. See Supplementary Figure 3 and Supplementary Table 3 for the distribution of answers on *current* *weight control* including all observations across waves.

In hindsight, the item *current weight control* may have been a viable option to use as a dependent variable as well. This item measured whether individuals are trying to gain weight, thus including participants that perceive themselves as underweight. In the dieting variable that was used in the analyses, it was only asked whether one dieted to lose or maintain weight. Then again, the variable current weight control may be less reliable, as it measures dieting in one particular moment only. There were indeed good reasons to choose the dieting variable currently included in the analyses. However, future research could include both variables in the study and further examine how the results for both measures compare.

**Supplementary Text 2. Results**

**Descriptive Statistics**

See Supplementary Table 4 for the descriptive statistics of the main study variables per wave and their intercorrelations. See Supplementary Table 5 for the descriptive statistics of the entire sample (including all waves) per gender. Supplementary Table 6 shows the Pearson’s product-moment correlations between the main variables within each time point.

**Gender differences**

With respect to the proportion scores of the sociometric nominations, *t*-tests including all observations across waves indicated significant gender differences for popularity, *t*(3065.3) = 6.700, *p* < .001, unpopularity, *t*(3184.7) = -2.226, *p* = 0.026, liking, *t* (3173.5) = -7.524, *p* < .001, and disliking, *t*(2988.1) = 5.556, *p* < .001. Thus, males received more nominations for most popular and least liked than females. Females received more nominations for most liked and least popular than males. Shortly said, males are more likely to be popular and females are more likely to be liked.

When doing the *t*-test per wave, gender differences were still significant for popularity, thus males receive more most popular nominations than females. For unpopularity, gender differences were only significant at T3, *t*(1056)= -2.532, *p* = 0.011. Thus, only at T3, females were more unpopular than males. For liking and disliking, gender differences were still significant at T1 and T2, thus females received more most liked nominations than males and males received more least liked nominations than females, but not at T3. A *t*-test with self-esteem indicated that males had a significantly higher self-esteem than females, *t*(2877.3) = 18.55, *p* < .001, which was still the case when doing the analysis per wave.

A chi-square test with binary weight perception indicated that the weight perception difference between genders was significant, *χ*2(1) = 21.585, *p* < .001. Thus, the weight perception of females was more negative than of males. A chi-square test with binary dieting indicated that females significantly dieted more often than males, *χ*2(1) = 220.47, *p* < .001. The same conclusions were drawn when doing the chi-square tests and t-tests with the observations separately per wave.

**Correlations Between Main Study Variables**

Supplementary Table 4 shows the stability of the variables, computed as Pearson’s correlations over time using Hmisc in R (Harrel, & Dupont, 2018). As can be seen, all variables were significantly correlated between waves. Stabilities were quite high for popularity, unpopularity, and self-esteem. The stabilities of dieting, weight perception, liking, and disliking were somewhat lower. Notably, the lowest correlations were seen for liking at T3, which was not highly correlated to liking at T1 and at T2.

**Supplementary Table 1**

*Sample Sizes by Wave and Grade*

|  | Grade | | | |  |
| --- | --- | --- | --- | --- | --- |
| Year | 1 | 2 | 3 | 4 | Total |
| 2013 | 259 | 282 | 285 | 246 | 1072 |
| 2014 | 286 | 260 | 293 | 247 | 1086 |
| 2015 | 273 | 283 | 277 | 250 | 1083 |
| Total | 818 | 825 | 855 | 743 | 3241 |

**Supplementary Table 2**

*Descriptive Statistics of the Dependent Variables per Wave*

| Variable | Wave | *n* | *M* | *SD* | Minimum | Maximum |
| --- | --- | --- | --- | --- | --- | --- |
| Weight perception | 1 | 1071 | 0.09 | 0.59 | -2 | 2 |
| (all categories) | 2 | 1086 | 0.11 | 0.55 | -2 | 2 |
|  | 3 | 1083 | 0.08 | 0.53 | -2 | 2 |
| Weight perception | 1 | 1071 | 0.27 | 0.45 | 0 | 1 |
| (binary) | 2 | 1086 | 0.27 | 0.44 | 0 | 1 |
|  | 3 | 1083 | 0.24 | 0.43 | 0 | 1 |
| Dieting (five | 1 | 1071 | 0.50 | 0.99 | 0 | 4 |
| categories) | 2 | 1086 | 0.47 | 0.91 | 0 | 4 |
|  | 3 | 1083 | 0.43 | 0.89 | 0 | 4 |
| Dieting (binary) | 1 | 1071 | 0.28 | 0.45 | 0 | 1 |
|  | 2 | 1086 | 0.28 | 0.45 | 0 | 1 |
|  | 3 | 1083 | 0.26 | 0.44 | 0 | 1 |

**Supplementary Table 3**

*Frequencies of Current Weight Control*

|  |  | Frequency | Percent | Valid percent | Cumulative percent |
| --- | --- | --- | --- | --- | --- |
| Valid | No | 1331 | 41,1 | 43,6 | 43,6 |
|  | Yes, I am trying to lose weight | 680 | 21,0 | 22,3 | 65,9 |
|  | Yes, I am trying to gain weight | 180 | 5,6 | 5,9 | 71,8 |
|  | Yes, I am trying to maintain the same weight | 861 | 26,6 | 28,2 | 100,0 |
|  | Total | 3052 | 94,2 | 100,0 |  |
| Missing | System | 188 | 5,8 |  |  |
| Total |  | 3240 | 100,0 |  |  |

*Note.* Answers to the question: “Are you trying to do something about your weight right now?”

**Supplementary Table 4**

*Descriptive Statistics of the Main Variables Per Wave and Intercorrelations*

|  |  |  |  |  |  |  | Intercorrelations | | |
| --- | --- | --- | --- | --- | --- | --- | --- | --- | --- |
| Variable | Wave | *n* | *M* | *SD* | Minimum | Maximum | T1 | T2 | T3 |
| Popularity | 1 | 1071 | 0.18 | 0.24 | 0.00 | 0.96 | - | .72 | .62 |
|  | 2 | 1086 | 0.16 | 0.23 | 0.00 | 0.95 | .72 | - | .73 |
|  | 3 | 1083 | 0.15 | 0.21 | 0.00 | 0.93 | .62 | .73 | - |
| Unpopularity | 1 | 1071 | 0.15 | 0.23 | 0.00 | 0.96 | - | .72 | .63 |
|  | 2 | 1086 | 0.13 | 0.22 | 0.00 | 1.00 | .72 | - | .63 |
|  | 3 | 1083 | 0.13 | 0.21 | 0.00 | 1.00 | .63 | .63 | - |
| Liking | 1 | 1071 | 0.18 | 0.10 | 0.00 | 0.63 | - | .42 | .15 |
|  | 2 | 1086 | 0.16 | 0.10 | 0.00 | 0.53 | .42 | - | .24 |
|  | 3 | 1083 | 0.15 | 0.08 | 0.00 | 0.48 | .15 | .24 | - |
| Disliking | 1 | 1071 | 0.09 | 0.11 | 0.00 | 0.79 | - | .52 | .37 |
|  | 2 | 1086 | 0.07 | 0.11 | 0.00 | 0.70 | .52 | - | .45 |
|  | 3 | 1083 | 0.07 | 0.10 | 0.00 | 0.76 | .37 | .45 | - |
| Self-esteem | 1 | 1071 | 3.07 | 0.54 | 1.00 | 4.00 | - | .66 | .61 |
|  | 2 | 1086 | 3.12 | 0.52 | 1.30 | 4.00 | .66 | - | .66 |
|  | 3 | 1083 | 3.18 | 0.55 | 1.10 | 4.00 | .61 | .66 | - |
| Dieting^a^ | 1 | 1071 | 0.28 | 0.45 | 0.00 | 1.00 | - | .44 | .31 |
|  | 2 | 1086 | 0.28 | 0.45 | 0.00 | 1.00 | .44 | - | .54 |
|  | 3 | 1083 | 0.26 | 0.44 | 0.00 | 1.00 | .31 | .54 | - |
| Weight | 1 | 1071 | 0.27 | 0.45 | 0.00 | 1.00 | - | .49 | .41 |
| Perception^a^ | 2 | 1086 | 0.27 | 0.44 | 0.00 | 1.00 | .49 | - | .51 |
|  | 3 | 1083 | 0.24 | 0.43 | 0.00 | 1.00 | .41 | .51 | - |

*Note*. ^a^Weight perception and dieting with binary outcomes. All correlations were significantly different from zero (*p* < .001).

**Supplementary Table 5**

*Desciptive statistics of the entire dataset per gender*

| Variable | Gender | *n* | *M* | *SD* | Minimum | Maximum | *t*-test | *χ*^2^ |
| --- | --- | --- | --- | --- | --- | --- | --- | --- |
| Popularity | Males | 1563 | 0.19 | 0.24 | 0.00 | 0.96 | 6.70*** |  |
|  | Females | 1629 | 0.14 | 0.20 | 0.00 | 0.95 |  |  |
| Unpopularity | Males | 1563 | 0.13 | 0.21 | 0.00 | 1.00 | -2.23* |  |
|  | Females | 1629 | 0.15 | 0.23 | 0.00 | 1.00 |  |  |
| Liking | Males | 1563 | 0.15 | 0.09 | 0.00 | 0.46 | -7.52*** |  |
|  | Females | 1629 | 0.18 | 0.10 | 0.00 | 0.63 |  |  |
| Disliking | Males | 1563 | 0.09 | 0.12 | 0.00 | 0.78 | 5.56*** |  |
|  | Females | 1629 | 0.07 | 0.09 | 0.00 | 0.79 |  |  |
| Self-esteem | Males | 1563 | 3.30 | 0.48 | 1.30 | 4.00 | 18.55*** |  |
|  | Females | 1629 | 2.95 | 0.54 | 1.00 | 4.00 |  |  |
| Dieting (original | Males | 1563 | 0.24 | 0.68 | 0 | 4 |  |  |
| answers) | Females | 1629 | 0.69 | 1.07 | 0 | 4 |  |  |
| Dieting (binary) | Males | 1563 | 0.15 | 0.36 | 0 | 1 |  | 220.47*** |
|  | Females | 1629 | 0.39 | 0.49 | 0 | 1 |  |  |
| Weight perception | Males | 1563 | 0.00 | 0.51 | -2 | 2 |  |  |
| (original answers) | Females | 1629 | 0.18 | 0.59 | -2 | 2 |  |  |
| Weight Perception | Males | 1563 | 0.22 | 0.42 | 0 | 1 |  | 21.56*** |
| (binary) | Females | 1629 | 0.30 | 0.46 | 0 | 1 |  |  |

*Note*. *** *p* < .001.

**Supplementary Table 6**

*Bivariate Cross-sectional Pearson Correlations between the Main Study Variables*

|  |  | 1 | 2 | 3 | 4 | 5 | 6 | 7 | 8 |
| --- | --- | --- | --- | --- | --- | --- | --- | --- | --- |
| 1. Popularity | T1 |  |  |  |  |  |  |  |  |
|  | T2 |  |  |  |  |  |  |  |  |
|  | T3 |  |  |  |  |  |  |  |  |
| 2. Unpopularity | T1 | -.42*** |  |  |  |  |  |  |  |
|  | T2 | -.38*** |  |  |  |  |  |  |  |
|  | T3 | -.37*** |  |  |  |  |  |  |  |
| 3. Liking | T1 | .28*** | -.41*** |  |  |  |  |  |  |
|  | T2 | .22*** | -.37*** |  |  |  |  |  |  |
|  | T3 | .25*** | -.32*** |  |  |  |  |  |  |
| 4. Disliking | T1 | .05 | .37*** | -.35*** |  |  |  |  |  |
|  | T2 | .07* | .38*** | -.33*** |  |  |  |  |  |
|  | T3 | .10*** | .27*** | -.31*** |  |  |  |  |  |
| 5. Self-esteem | T1 | .01 | -.06 | .02 | -.02 |  |  |  |  |
|  | T2 | .03 | -.09** | .00 | -.02 |  |  |  |  |
|  | T3 | .08* | -.07* | .02 | -.05 |  |  |  |  |
| 6. Gender | T1 | -.09** | .01 | .16*** | -.12*** | -.33*** |  |  |  |
|  | T2 | -.13*** | .03 | .19*** | -.12*** | -.36*** |  |  |  |
|  | T3 | -.13*** | .08* | .04 | -.06 | -.30*** |  |  |  |
| 7. Weight Perception | T1 | -.04 | .06 | -.09** | .04 | -.25*** | .09** |  |  |
|  | T2 | -.02 | .05 | .00 | .04 | -.30*** | .09** |  |  |
|  | T3 | -.15*** | .07* | -.05 | .01 | -.28*** | .08* |  |  |
| 8. Dieting | T1 | .01 | .05 | -.03 | .04 | -.27*** | .27*** | .24*** |  |
|  | T2 | .00 | .05 | .05 | .00 | -.36*** | .31*** | .27*** |  |
|  | T3 | -.01 | .04 | .01 | .01 | -.32*** | .22*** | .26*** |  |

*Note*. Weight perception and dieting with binary outcomes. Only cross-sectional correlations.

* *p* < .05. ** *p* < .01. *** *p* < .001.

| **Supplementary Fig. 1**  *Barplots of the distribution of weight perception (including all observations across the three waves) with the three different types of answer categories* | | | | |
| --- | --- | --- | --- | --- |
| 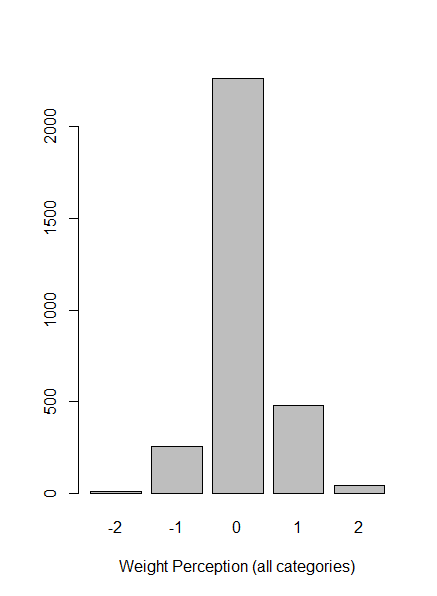 | 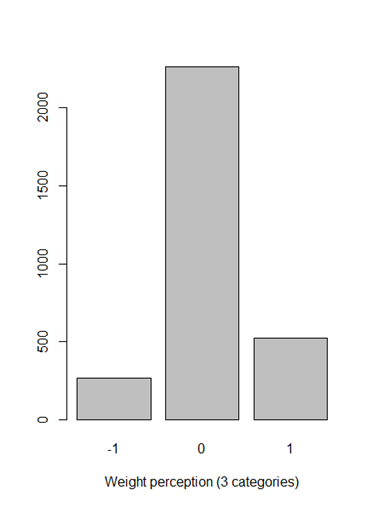 | | 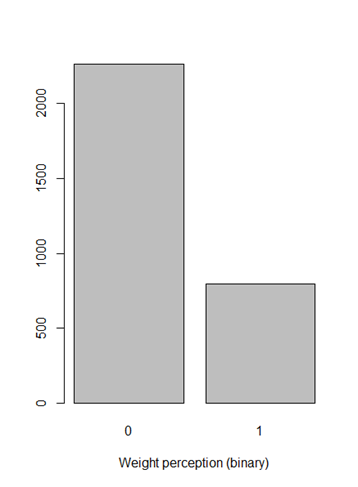 | |
| *Note*. The score 0 always indicates perception of having the right weight. In the left and central panel, the negative scores indicate feeling underweight and the positive scores indicate feeling overweight. In the panel on the right, the score 1 indicates feeling either underweight or overweight. | | | | |
| **Supplementary Fig. 2**  *Barcharts of the frequency of answers regarding dieting in the past year including all observations across the three waves, where 0 indicates “never” for both panels* | | | |  |
| 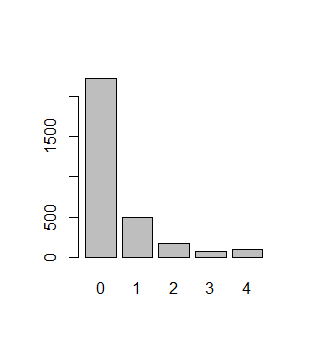 | | 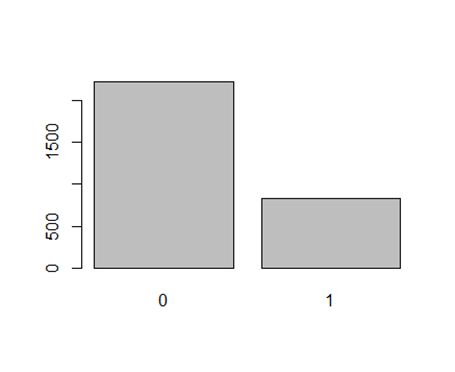 | |  |
| *Note*. *In the left panel*: 1 =“1-2 times”, 2 = “3-4 times”, 3 = “5-6 times” and 4 = “7 times or more”. *In the right panel*: 1 = “once or more”. | | | |  |

**Supplementary Fig. 3**

*Barchart of the frequency of answers regarding weight control in the present including all observations across the three waves*


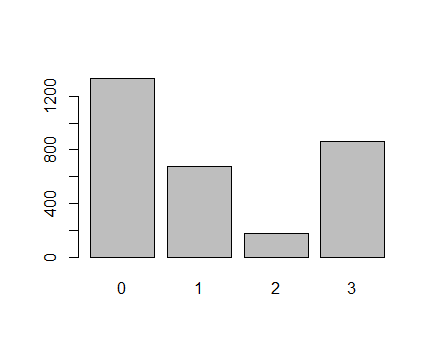


*Note*. 0 = “no”, 1 = “yes, I am trying to lose weight”, 2 = “yes I am trying to gain weight”, 3 = “yes, I am trying to maintain the same weight”.
